# Bone Marker Proteins at Baseline and After Insulin-Induced Hypoglycaemia in Type 2 Diabetes

**DOI:** 10.3390/ijms262311432

**Published:** 2025-11-26

**Authors:** Benjamin M. L. Atkin, Thozhukat Sathyapalan, Laura Dempsey, Stephen L. Atkin, Alexandra E. Butler

**Affiliations:** 1 Edinburgh Royal Infirmary, Old Dalkeith Road, Edinburgh EH16 4SA, UK; ben.atkin@nhs.scot; 2 Academic Endocrinology, Diabetes and Metabolism, Hull York Medical School, Hull YO10 5DD, UK; thozhukat.sathyapalan@hyms.ac.uk; 3 Data Science Centre, School of Population Health, Royal College of Surgeons in Ireland (RCSI), D02 YN77 Dublin, Ireland; lauradempsey@rcsi.ie; 4 Research Department, Royal College of Surgeons in Ireland, Bahrain, Adliya P.O. Box 15503, Bahrain; satkin@rcsi.com

**Keywords:** type 2 diabetes, bone markers, hypoglycaemia, Dickkopf-related protein 1 (DKK1), cathepsin

## Abstract

Type 2 diabetes (T2D) is associated with normal or higher bone mineral density (BMD), but there is a higher fracture rate. Hypoglycaemia does not affect BMD but may cause fractures directly through falls and may affect bone cellular metabolism. We examined circulating bone marker proteins (BMPs) in response to induced hypoglycaemia in T2D versus controls. A prospective exploratory parallel study design was conducted in T2D patients (*n* = 23) and healthy controls (*n* = 23) who underwent blood SOMAscan proteomic analysis of bone biomarkers at baseline, hypoglycaemia, and post-hypoglycaemia time points. Unadjusted repeated measures linear mixed modeling was used for analysis. Linear mixed modeling of the proteins showed that the way most BMPs changed over time did not differ between groups. At baseline, Dickkopf-related protein 1 (DKK1), cathepsin A, cathepsin S, and cathepsin Z were increased in T2D versus controls (*p* < 0.05), whilst fibroblast growth factor 23 (FGF23) was lower in T2D versus controls (*p* ≤ 0.05). Following hypoglycemia, transient changes from baseline occurred in DKK1, cathepsin A, cathepsin G, cathepsin H, cathepsin S, cathepsin Z, parathyroid hormone (PTH), Sphingosine kinase 1 and 2 (SPK1/2), and interleukin-1 beta (IL1 beta) over the post-hypoglycaemia time course. There was decreased cathepsin S in T2D from baseline to 24 h compared to the control group, and increased cathepsin Z at 24 h for both groups overall compared to baseline (*p* < 0.05). Baseline-raised cathepsins (A, S, Z) in T2D may enhance osteoclastic resorption, whilst raised DKK1 could inhibit osteoblast differentiation and suppress bone formation. Hypothetically, this may lead to a decline in bone quality through a resorption-enhanced, low bone formation imbalance. The effects of hypoglycaemia on bone physiology appear to extend significantly beyond the initial insult, as seen for cathepsin S and Z, which differed at 24 h compared to baseline.

## 1. Introduction

Type 2 diabetes mellitus (T2D) is associated with a normal or raised bone mineral density (BMD) compared to non-diabetic individuals, due to obesity, with higher body mass index (BMI) causing greater mechanical loading on bones [1]. However, despite this apparent increase in bone quantity, individuals with T2D face a significantly heightened risk of fragility fractures, indicating a potential discrepancy between bone density and bone quality [2,3]. Extensive epidemiological studies demonstrate a 40–70% increased risk of hip fractures in individuals with T2D, a rate comparable between men and women, with vertebral and ankle fractures disproportionately represented [4,5].

Several mechanisms potentially contribute to the deterioration of bone quality in T2D. Chronic hyperglycaemia promotes the accumulation of advanced glycation end-products (AGEs) in type I collagen, adversely affecting its cross-linking and compromising the bone matrix’s overall toughness and integrity [3,6]. Moreover, hyperglycaemia may impair osteoblast activity by inducing oxidative stress and exacerbating insulin resistance, thereby reducing bone formation [3,7]. Conversely, osteoclast function might also be impaired, suggesting a multifactorial influence on bone remodeling and turnover [3,8]. Differentiation of mesenchymal stem cells within the bone marrow may increasingly favor adipocyte formation over osteoblastogenesis, culminating in greater bone marrow adiposity that can impede bone health [9].

Microvascular complications associated with diabetes, such as diabetic microangiopathy, can impair nutrient and oxygen delivery to bone, affecting both the remodeling and repair processes [10]. The long-term use of certain anti-diabetic medications, particularly thiazolidinediones (TZDs), has been linked to an increased fracture risk. TZDs are associated with progressive bone loss, which compromises skeletal integrity [11,12]. Insulin therapy has also been implicated in higher fracture incidence, predominantly through the risk of hypoglycaemia-induced falls [13]. Conversely, metformin may confer skeletal benefits through activation of adenosine monophosphate (AMP)-activated protein kinase (AMPK) signaling [14].

Hypoglycaemia is a common complication of diabetes therapy, particularly in individuals treated with insulin or insulin secretagogues such as sulfonylureas; however, hypoglycaemia does not impact BMD, but bone fractures may result from falls [15]. Acute hypoglycaemia triggers oxidative stress, which may temporarily impair osteoblast function and survival, potentially compromising skeletal integrity [16]. It has been reported that, in those with both type 1 diabetes and insulin-treated subjects with T2D, recurrent hypoglycaemia has been associated with an increased fracture rate, primarily due to falls [17], suggesting that an additional mechanism may be long-term skeletal effects due to chronic hypoglycaemia compromising bone that may then fracture more easily with a fall [18]. Therefore, this study was undertaken to determine whether changes in bone marker proteins (BMPs) following hypoglycaemia may contribute to increased bone fragility, with the null hypothesis that BMPs would not differ.

## 2. Results

### 2.1. Demographic and Biochemical Characteristics of Study Participants

T2D patients were significantly older (64 versus 60 years, *p* < 0.0001), had higher glycated haemoglobin (Haemoglobin A1c, HbA1c) (6.8 versus 5.6%, *p* < 0.0001), body weight (91 versus 80 kg, *p* < 0.0001), and body mass index (BMI) (32 versus 28 kg/m^2^, *p* < 0.0001) compared to controls (Table 1). Systolic blood pressure (SBP) and diastolic BP (DBP) were elevated in T2D compared to controls; 132 versus 122 mmHg, *p* = 0.001, and 81 versus 75 mmHg, *p* = 0.003. T2D patients had significantly lower total cholesterol (42 versus 48 mmol/L, *p* = 0.014) and high density lipoprotein (HDL) cholesterol (1.1 versus 1.5 mmol/L, *p* = 0.001), while triglycerides and low density lipoprotein (LDL) cholesterol levels were similar (*p* > 0.05). C-reactive protein (CRP), as a measure of inflammation, did not differ (*p* > 0.05).

### 2.2. Linear Mixed Model for Repeated Measures for Each Bone Marker Protein Between T2D and Controls at Baseline

**Figure 1:**
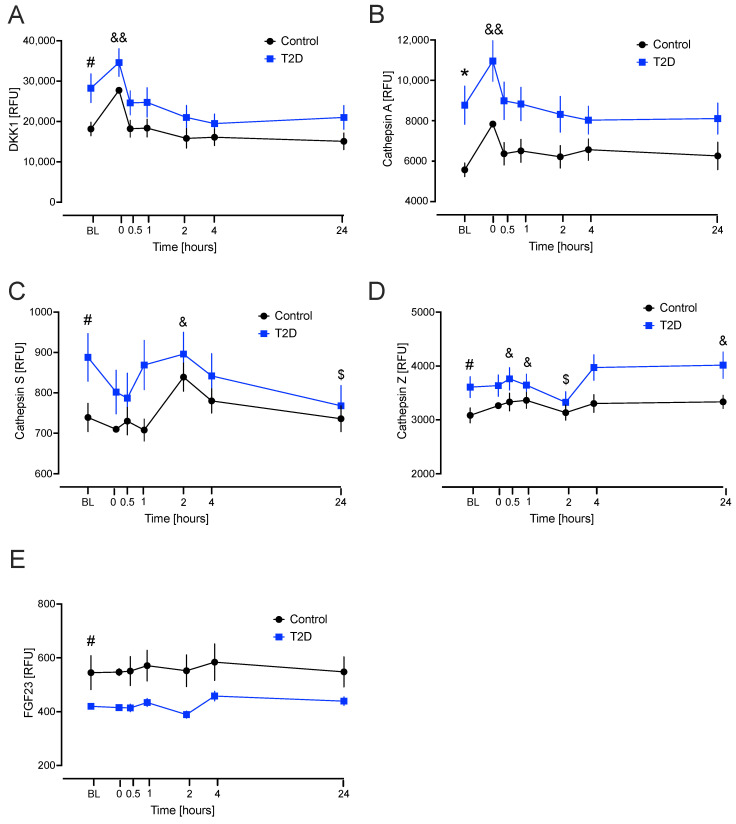
Significant changes in bone marker proteins in plasma that differed at baseline between type 2 diabetes (T2D) and control cohorts. Blood sampling was performed at baseline (BL), at hypoglycaemia (0 min), and post-hypoglycaemia (30 min, 1, 2, 4 and 24 h) for controls (black circles) and for T2D (blue squares). At BL, blood sugar (BS) was 7.5 ± 0.4 mM (for T2D) and 5.0 ± 0.1 mM (for control, C). At the point of hypoglycaemia, blood sugar (BS) was 2.0 ± 0.03 mM (for T2D) and 1.8 ± 0.05 mM (for control). (**A**) Dickkopf-related protein 1 (DKK1); (**B**) cathepsin A; (**C**) cathepsin S; (**D**) cathepsin Z; (**E**) fibroblast growth factor 23 (FGF23). Statistics: # *p* < 0.05 and * *p* < 0.01, comparison between T2D vs. Control. $ *p* < 0.05 comparison from baseline in T2D; & *p* < 0.05 and && *p* < 0.01, comparison from baseline for combined groups. RFU, Relative fluorescent unit.

Dickkopf-related protein 1 (DKK1): the model showed that the level in the T2D group was significantly higher than the control group at baseline (*p* = 0.017) (Figure 1A). Cathepsin A: the model showed that the level in the T2D group was significantly higher at baseline compared to the control group (*p* = 0.004) (Figure 1B). Cathepsin S: the model showed that the level in the T2D group was significantly higher at baseline compared to the control group (*p* = 0.027) (Figure 1C). Cathepsin Z: the model showed that the level in the T2D group was significantly higher at baseline compared to the control group (*p* = 0.032). Fibroblast growth factor 23 (FGF23): the model showed that the level in the T2D group was significantly lower than the control group at baseline (*p* = 0.026) (Figure 1E) (Table 2).

### 2.3. Linear Mixed Model for Repeated Measures for Each Bone Marker Protein from Baseline to the Hypoglycaemia Time Points

The linear mixed model for repeated measures approach allows us to evaluate both overall trends (combined groups) and group-specific effects. In most instances, it was observed that the way in which BMP markers changed over time did not differ between groups. DKK1: compared to baseline, there was a significant increase in the level at hypoglycaemia (*p* = 0.001) overall (combined groups). Cathepsin A: there was a significant increase in the level at hypoglycaemia (*p* = 0.003) compared to baseline overall (combined groups). Cathepsin S: Compared to baseline, there was a significant increase in the level at 2 h (*p* = 0.01) overall (combined groups). Additionally, there was a significant decrease in the level in the T2D group compared to the control group from baseline to 24 h (*p* = 0.049). Cathepsin Z: compared to baseline, there was a significant increase in the level at 30 min (*p* = 0.047), 1 h (*p* = 0.022), and 24 h (*p* = 0.044), overall (combined groups); there was a significant decrease in the level in the T2D group compared to the control group from baseline to 2 h (*p* = 0.016); FGF23 did not differ significantly over time (Figure 1).

**Figure 2:**
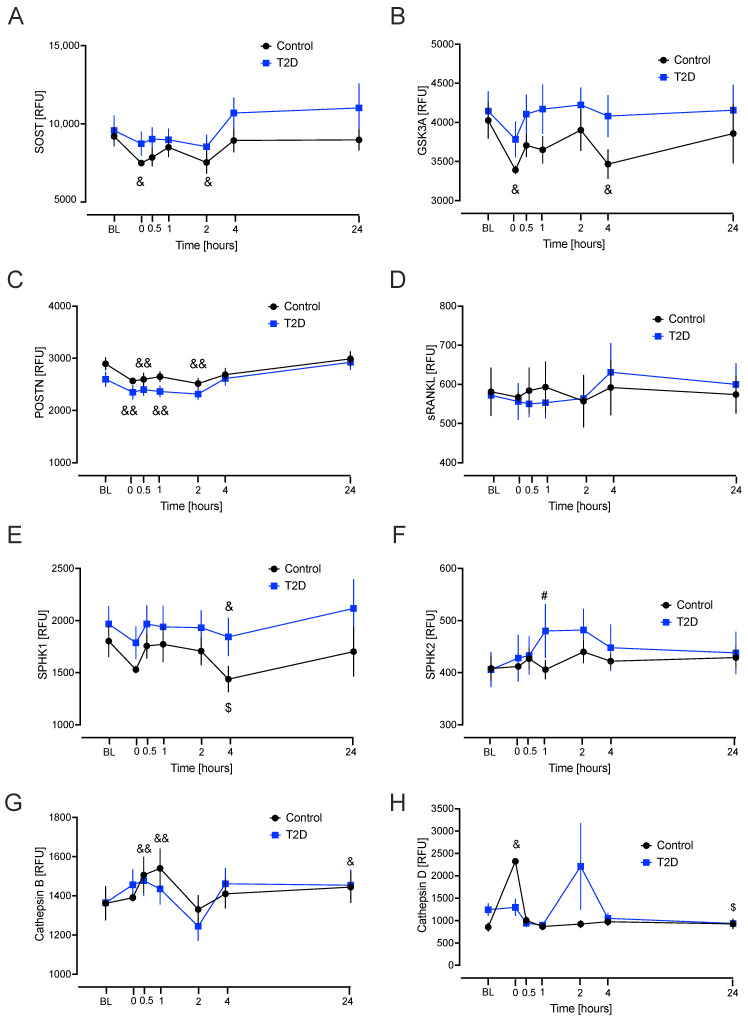
Significant changes in bone marker proteins in plasma that differed in response to hypoglycaemia from baseline in type 2 diabetes (T2D) and control cohorts. Blood sampling was performed at baseline (BL), at hypoglycaemia (0 min), and post-hypoglycaemia (30 min, 1, 2, 4, and 24 h) for controls (black circles) and for T2D (blue squares). At BL, blood sugar (BS) was 7.5 ± 0.4 mM (for T2D) and 5.0 ± 0.1 mM (for control, C). At the point of hypoglycaemia, blood sugar (BS) was 2.0 ± 0.03 mM (for T2D) and 1.8 ± 0.05 mM (for control). (**A**) Sclerostin (SOST); (**B**) Glycogen synthase kinase-3 alpha/beta (GSK3A); (**C**) Periostin (POSTN); (**D**) Tumor necrosis factor ligand superfamily member 11 (sRANKL); (**E**) Sphingosine kinase 1 (SPHK1); (**F**) Sphingosine kinase 2 (SPHK2); (**G**) cathepsin B; (**H**) cathepsin D. Statistics: # *p* < 0.05, comparison between T2D vs. Control. $ *p* < 0.05 comparison from baseline in T2D; & *p* < 0.05 and && *p* < 0.01, comparison from baseline for combined groups. RFU, Relative fluorescent unit.

Sclerostin: compared to baseline, there was a significant decrease in the level at hypoglycaemia (*p* = 0.029) and 2 h (*p* = 0.044) overall (combined groups). Glycogen synthase kinase-3 alpha/beta (GSK3): the model showed that, compared to baseline, there was a significant decrease in the level at hypoglycaemia (*p* = 0.023) and 4 h (*p* = 0.038) overall (combined groups). Periostin: the model showed that, compared to baseline, there was a significant decrease in the level at hypoglycaemia (*p* < 0.001), 30 min (*p* < 0.001), 1 h (*p* = 0.007), and 2 h (*p* < 0.001) overall (combined groups). Tumor necrosis factor ligand superfamily member 11 (sRANKL): the model showed no differences to baseline values. Sphingosine kinase 1 (SPK1): the model showed that, compared to baseline, there was a significant decrease in the level at 4 h (*p* = 0.04) overall (combined groups). Sphingosine kinase 2 (SPK2): the model showed that, compared to baseline, there was a significant increase in the level at 1 h post-hypoglycaemia (*p* = 0.011) in the T2D group compared to the control group. Cathepsin B: the model showed that, compared to baseline, there was a significant increase in the overall level at 30 min (*p* < 0.001), 1 h (*p* < 0.001), and 24 h (*p* = 0.03). Cathepsin D: the model showed that, compared to baseline, there was a significant increase at hypoglycaemia compared to baseline overall (combined groups) (*p* = 0.028) (Figure 2).

**Figure 3:**
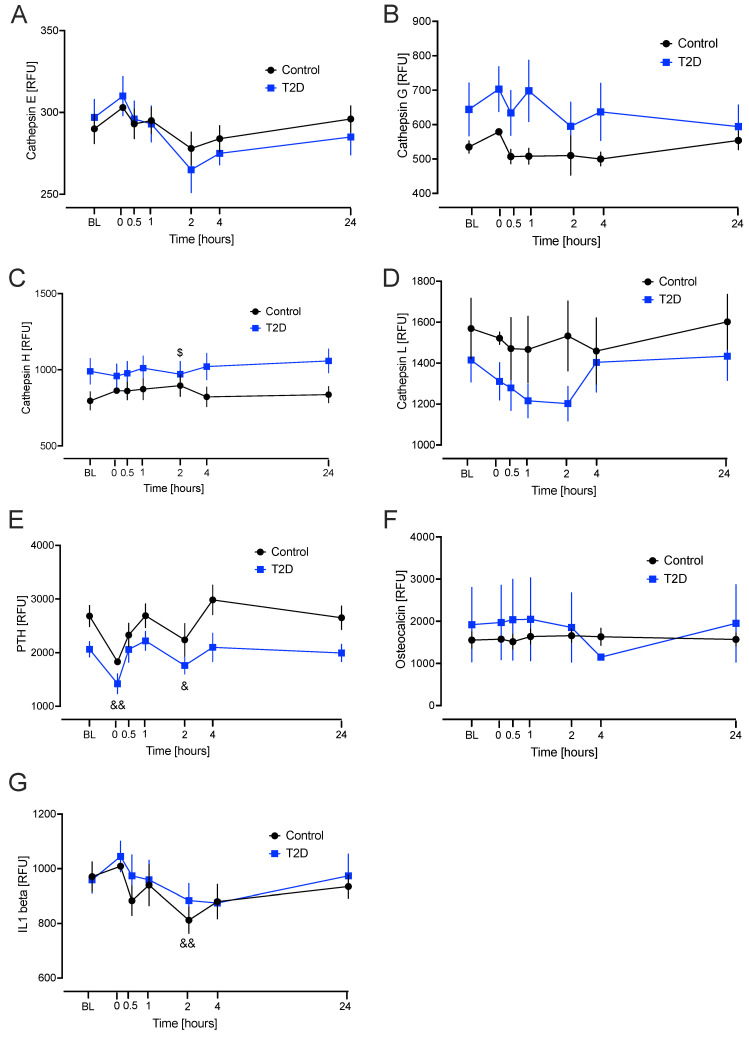
Significant changes in bone marker proteins in plasma that differed in response to hypoglycaemia from baseline in type 2 diabetes (T2D) and control cohorts. Blood sampling was performed at baseline (BL), at hypoglycaemia (0 min), and post-hypoglycaemia (30 min, 1, 2, 4, and 24 h) for controls (black circles) and for T2D (blue squares). At BL, blood sugar (BS) was 7.5 ± 0.4 mM (for T2D) and 5.0 ± 0.1 mM (for control, C). At the point of hypoglycaemia, blood sugar (BS) was 2.0 ± 0.03 mM (for T2D) and 1.8 ± 0.05 mM (for control). (**A**) cathepsin E; (**B**) cathepsin G; (**C**) cathepsin H; (**D**) cathepsin L; (**E**) parathyroid hormone (PTH); (**F**) osteocalcin; (**G**) Interleukin-1 beta (IL-1 beta). Statistics: $ *p* < 0.05 comparison from baseline in T2D; & *p* < 0.05 and && *p* < 0.01, comparison from baseline for combined groups. RFU, Relative fluorescent unit.

Cathepsin E: the model showed no differences from baseline values. Cathepsin G: the model showed no differences from baseline values. Cathepsin H: the model showed a significant decrease in the level from baseline to 2 h in the T2D group compared to the control group (*p* = 0.049). Cathepsin L: the model showed no differences from baseline values. Parathyroid hormone (PTH): the model showed that, compared to baseline, there was a significant decrease in the level at hypoglycaemia (*p* < 0.001) and 2 h (*p* = 0.042) overall (combined groups). Osteocalcin: the model showed no differences from baseline values. Interleukin-1 beta (IL1 beta): the model showed that, compared to baseline, there was a significant decrease in the level at 2 h (*p* = 0.004) overall (combined groups) (Figure 3).

The other BMPs showed no differences between cohorts at any point during the time course (Figure 2 and Figure 3).

## 3. Discussion

This study examines BMPs in T2D and control groups at baseline and following induction of severe hypoglycaemia with a 24 h follow-up period. We observed significant baseline differences between cohorts for several BMPs; in T2D, DKK1, cathepsin A, cathepsin S, and cathepsin Z were elevated, while FGF23 was decreased. Hypothetically, these data suggest that the BMPs that differed reflect an increased bone resorptive state that may impact bone fragility, suggesting they may contribute to the underlying molecular mechanisms; however, the data needs to be treated with caution as this was an exploratory study. This study is novel as there is scant data on these BMPs in diabetes-related bone physiology, and there is no study measuring all these BMPs at the same time, generating questions on how their combined potential activities may be affecting bone in T2D.

Receptor activator of nuclear factor-kB ligand (RANKL) increases bone resorption through osteoclast activation. It has been shown to be elevated in diabetes, where it has an additional association with glucose metabolism [19]. The changes seen for DKK1, cathepsin A, cathepsin S, cathepsin Z, and FGF23 that potentially favor a bone resorptive state may contribute to the resorptive effects of RANKL in diabetes. In T2D, there is the clinical paradox that, despite normal or elevated bone mineral density (BMD) in subjects with T2D, there is a significantly increased risk of fractures [20]. High-resolution imaging studies show that patients with T2D often have greater trabecular bone, consistent with higher BMD, but they remain at high risk of fracture, which could be partially explained by the greater porosity measured in their cortical bone [20].

T2D is associated with chronic low-grade inflammation, which promotes bone loss by inhibiting new bone formation and accelerating bone resorption, leading to increased fracture risk and diabetic bone disease [7]. In this study, there were no differences in either CRP or IL-1 beta, as markers of systemic inflammation, indicating that the changes reported here were unlikely to be due to confounding inflammation.

Cathepsin A (also known as the lysosomal protective protein) plays a critical role in stabilizing lysosomal enzymes such as β-galactosidase and neuraminidase-1, ensuring sustained lysosomal function in osteoclasts [21]. Elevated levels of cathepsin A may prolong osteoclast proteolytic efficiency, thereby promoting sustained bone resorption, but there is no literature on cathepsin A in diabetes bone disease. In this study, cathepsin A levels were higher in T2D compared to controls at baseline, but with comparable changes from baseline in response to hypoglycaemia in both groups.

Cathepsin S is a potent cysteine protease active at neutral pH, enabling it to act extracellularly in degrading collagen and elastin, and has been noted to be elevated in diabetes [22]. It is strongly associated with inflammatory matrix remodeling, including in rheumatoid arthritis and osteoporosis (preprint [23]), but there is no literature on cathepsin S in diabetes bone disease. Elevated cathepsin S could suggest increased extracellular matrix degradation that would be reflected in increasing bone fragility in inflammatory states. In this study, cathepsin S levels were higher in T2D compared to controls at baseline in accordance with the findings of others [22] and there was a significant decrease in the T2D group compared to the control group from baseline to 24 h, suggesting that the effects of hypoglycaemia appear to persist, with the result that, hypothetically, there may be some impairment in osteoclastic bone resorption.

Cathepsin Z is a cysteine protease distinguished by its RGD integrin-binding motif, which enhances osteoclast adhesion, migration, and bone resorption capacity [24]. Elevated levels of cathepsin Z are found in osteopenia and osteoporosis [24] and in diabetes [25], but there is no literature on cathepsin Z in diabetes bone disease. In this study, cathepsin Z levels were higher in T2D compared to controls at baseline in accordance with the literature [25], and there was a significant increase compared to baseline at 24 h (*p* = 0.044) when both groups were combined. This again suggests that the effects of hypoglycaemia may have prolonged effects on cathepsin Z, an increase in which may contribute to heightened bone resorptive activity.

In contrast to the cathepsin-mediated increase in resorption, DKK1 functions as a potent inhibitor of the Wnt/β-catenin pathway, a critical signaling cascade for osteoblast differentiation and bone formation [26]; elevated DKK1 suppresses osteoblastogenesis and bone formation and its role has been well-established in osteoporosis [27]. Elevation in DKK1 in T2D has been reported previously, in accordance with this study, when looking at cardiovascular risk indices [28].

FGF23 is a hormone that is mainly secreted by osteocytes and osteoblasts in bone, and FGF23 and its cofactor, Klotho, may play an independent role in directly regulating bone mineralization [29]. Parathyroid signaling in osteocytes increases the expression of FGF23 [30] and the elevated PTH may explain the elevation in FGF23 in this study.

Hypothetically, this overall BMP profile suggests an imbalance in which resorption is amplified by cathepsins and formation is suppressed by DKK1, leading to a deterioration of bone quality in the setting of T2D.

The linear mixed model showed that in most cases, the way in which the two groups changed over time did not differ. Transient changes following hypoglycaemia were seen for DKK1, cathepsin A, cathepsin B, cathepsin D, cathepsin S, cathepsin Z, sclerosin, GSK3, periostin, SPK 1 and 2, cathepsin H, PTH, and IL-1 beta: fluctuations were evident during the time course, such as for PTH, but larger studies would be needed to determine if was consistent. All BMPs but cathepsin S and Z returned to their respective baseline values by 4 h. In T2D, cathepsin S significantly decreased from baseline to 24 h compared to the control group. This suggests that the effects of hypoglycaemia on bone physiology last longer than the initial event and would suggest that cathepsins S and Z may continue to affect bone physiology even after 24 h. Further larger studies are warranted to confirm this finding; however, the other BMPs under study appeared not to have a prolonged effect on bone physiology in response to hypoglycaemia, and other mechanisms are likely responsible for possible adverse changes due to hypoglycaemia, such as oxidative stress [31], and may provoke sustained stress in osteocytes or bone marrow.

The potential clinical implication of this study is that those experiencing recurrent hypoglycaemic events, who are at risk of fractures due to falls, may be at enhanced risk of a fracture occurring due to disordered bone physiology through hypoglycaemia. Further definitive studies on BMPs and functional bone studies are needed to clarify this and perhaps identify preventative strategies. It is unknown whether the BMP parameters would have differed in this group in comparison to a group with a higher baseline HbA1c or whose diabetes was of longer duration; hypothetically, if no diabetes group experienced recurrent hypoglycaemia, the BMPs may not differ.

The strengths of this study include that the T2D subjects had a relatively short duration of disease with an average of 4.5 years and whose diabetes was well-controlled on conservative pharmacotherapy of metformin alone. As noted above, metformin may confer skeletal benefits through activation of AMPK signaling and support of osteoblast differentiation [14] that may have impacted the BMP levels; therefore baseline changes in BMPs cannot be definitively attributed to diabetes. In addition, the range of proteomic BMPs assessed included those involved in bone resorption and bone formation. However, given that this study examined plasma proteomics, it cannot be assumed that individual biomarkers are consistent with cellular levels or activity. Although clear differences in proteomic BMPs were observed between T2D and control cohorts, the study is limited by the small study number, and the data need to be treated with caution as this was an exploratory hypothesis-generating study. In addition, we cannot exclude that the time taken to achieve hypoglycaemia (62.8 min in T2D vs. 32.5 min in controls) may have had an impact on the BMP responses between T2D and controls. We acknowledge that adjusting for covariates in our repeated measures linear model would be preferable; however, our limited sample size constrained our ability to adjust for multiple covariates without compromising model stability and statistical power. Therefore, residual confounding cannot be excluded, and our results should be interpreted with caution. The bone turnover markers of C-terminal telopeptide of type 1 collagen (CTX), reflecting bone resorption, and procollagen type 1 N propeptide (P1NP), reflecting bone formation, differ in increased glycaemic variability [32], but were not measured in this study and would need to be included in the future. All subjects were Caucasian, and, therefore, these results may not be generalizable to other ethnic groups. Future larger studies with this BMP panel together with measures of BMD and bone strength are needed to determine their utility as biomarkers for bone physiology in T2D.

In conclusion, baseline-raised cathepsins (A, S, Z) in T2D may enhance osteoclastic resorption, while raised DKK1 could inhibit osteoblast differentiation and suppress bone formation. Hypothetically, this may potentially lead to a decline in bone quality through a resorption-enhanced, low bone formation imbalance. The effects of hypoglycaemia appear to last longer on bone physiology than changes at the point of hypoglycaemia such as is seen for cathepsins S and Z that differed at 24 h compared to baseline.

## 4. Materials and Methods

### 4.1. Study Design

This prospective, parallel-group study enrolled 46 participants, comprising 23 adults with T2D and 23 non-diabetic control subjects, as previously described [33]. The study was conducted at the Diabetes Center, Hull Royal Infirmary between 1 March 2017 and 10 January 2018. All participants provided written informed consent. Ethical approval was granted by the Northwest–Greater Manchester East Research Ethics Committee (REC number: 16/NW/0518) on 1 February 2017, and the trial was registered at ClinicalTrials.gov (NCT03102801) on 6 December 2016. The study was performed in accordance with the principles of the Declaration of Helsinki by Dr Sathyapalan. Eligible participants were Caucasian men and women aged 40–70 years, with a body mass index (BMI) between 18 and 49 kg/m^2^, normal renal and hepatic biochemistry, and no history of malignancy. Exclusion criteria included any contraindication to insulin infusion for induction of hypoglycaemia, such as ischemic heart disease, epilepsy, seizure history, drop attacks, adrenal insufficiency, or treated hypothyroidism. For the T2D cohort, inclusion required a diabetes duration of less than 10 years, treatment with a stable dose of metformin alone for at least three months, glycated hemoglobin (HbA1c) < 10% (86 mmol/mol), and no history of hypoglycaemic unawareness or hypoglycaemia within the preceding three months. Control participants underwent a standard oral glucose tolerance test to exclude diabetes.

### 4.2. Biochemical Markers

Blood samples were processed within 30 min of collection. Plasma was separated by centrifugation at 2000× *g* for 15 min at 4 °C, and aliquots were stored at −80 °C until batch analysis. Fasting plasma glucose (FPG), total cholesterol, triglycerides, and high-density lipoprotein (HDL) cholesterol were quantified using enzymatic methods on a Beckman AU 5800 analyzer (Beckman-Coulter, High Wycombe, UK).

### 4.3. Insulin Infusion

Participants underwent a standardized insulin infusion protocol as previously described. Following an overnight fast, intravenous cannulas were inserted into both antecubital fossae to facilitate simultaneous blood sampling and insulin administration. At 08:30 a.m., a continuous infusion of soluble insulin (Humulin S, Lilly, Basingstoke, UK) was initiated at 2.5 mU/kg/min. The infusion rate was increased by 2.5 mU/kg/min every 15 min until capillary blood glucose reached ≤2.2 mmol/L (≤40 mg/dL), or a single measurement of ≤2.0 mmol/L (36 mg/dL) was achieved. The mean time to hypoglycaemia differed significantly between groups: 62.8 ± 29.2 min in participants with T2D compared with 32.5 ± 15.1 min in controls. In the T2D group, blood glucose was first stabilized at 5.0 mmol/L (90 mg/dL) prior to induction of hypoglycaemia. Blood samples were collected at baseline, at the nadir of hypoglycaemia, and during recovery at 30 min, 1 h, 2 h, 4 h, and 24 h post-hypoglycaemia. Once hypoglycaemia was confirmed, participants were treated with an intravenous bolus of 150 mL 10% dextrose, and glucose concentrations were monitored every 5 min until recovery. The final 24 h blood sample was obtained at a follow-up visit the next day.

### 4.4. SOMAscan Assay

**Figure 4:**
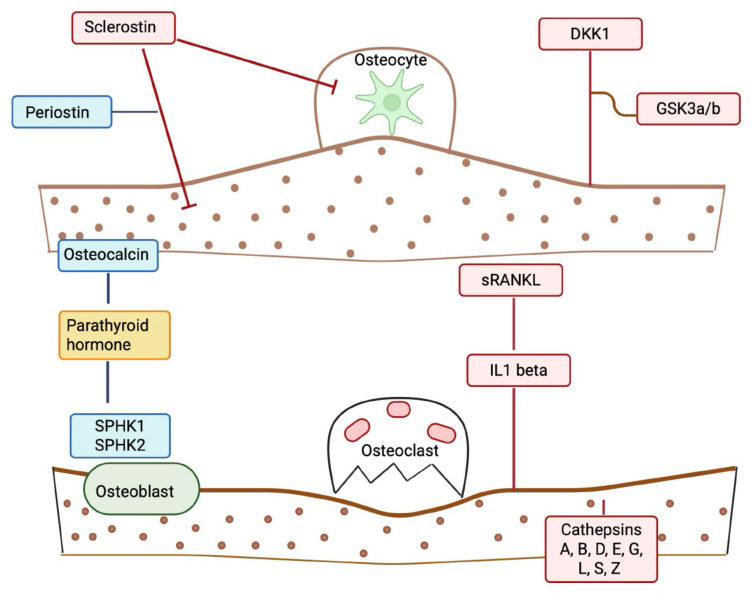
A schematic to illustrate the effects of the bone marker proteins on bone metabolism. Proteins depicted in blue are anabolic/formation factors. Proteins depicted in red are catabolic/formation factors. The protein in yellow (parathyroid hormone (PTH)) is a mixed regulatory factor. Straight lines indicate a stimulatory effect; blunt-ended lines indicate an inhibitory effect. The illustration was created using BioRender.com (with publication license).

The SOMAscan assay used to quantify proteins was performed on an in-house Tecan Freedom EVO liquid handling system (Tecan Group, Maennedorf, Switzerland) utilizing buffers and SOMAmers from the SOMAscan HTS Assay 1.3K plasma kit (SomaLogic, Boulder, CO, USA) according to the manufacturer’s instructions and as described previously [34]. Plasma samples were diluted and incubated with streptavidin-coated beads immobilized with dilution-specific SOMAmers, which bind to target proteins via photocleavable linkers. Following washing, bound proteins were biotinylated, released via photocleavage, and hybridized onto microarray chips. Fluorescence intensities were measured, and data were normalized using the SOMAscan software (version 3.1) pipeline. We used version 3.1 of the SOMAscan Assay, specifically targeting those BMPs in the SOMAscan panel. BMPs included Sclerostin, Dickkopf-related protein-1, Glycogen synthase kinase-3 alpha/beta, Periostin, Tumor necrosis factor ligand superfamily member 11, fibroblast growth factor 23, Sphingosine kinase 1, Sphingosine kinase 2, cathepsins A, B, D, E, G, H, L, S, and Z, parathyroid hormone, osteocalcin, and Interleukin-1 beta (*n* = 20) (Figure 4).

### 4.5. Statistics

No published studies are available that could allow for performing a power calculation; therefore, we undertook a hypothesis-generating exploratory pilot study. Power and sample size for pilot studies has been reviewed by Birkett and Day [35] who concluded that a minimum of 20 degrees of freedom was required to estimate effect size and variability. Hence, we recruited 23 patients to allow for dropouts. Data distributions were visually and statistically assessed for normality using the Kolmogorov–Smirnov (K-S) statistical test. To explore initial between group differences, Student’s t-test was used to compare differences between groups. Parametric analyses (independent t-tests) were applied to normally distributed variables, while non-parametric comparisons (Mann–Whitney U tests) were used for variables that did not meet normality assumptions. To explore any potential interactions between group and time, a linear mixed model for repeated measures was run for each BMP. Each BMP was used as the dependent variable, with treatment group, time point, and group-by-time interaction included as fixed effects and subject as a random effect. This approach accounts for individual variation and evaluates both overall trends and group-specific effects. A significant group-by-time interaction would indicate that groups differ in how BMP levels change over time. A *p* value of 0.05 or less was taken to be statistically significant. For statistical analysis, Graphpad Prism v10.4.1 (San Diego, CA, USA) and R Studio version 4.4.2. were utilized.

## Figures and Tables

**Table 1:** Demographic and clinical characteristics of the study participants. Data are presented as mean ± SD.

Baseline	T2D (*n* = 23)	Controls (*n* = 23)	*p* Value
Age (years)	64 ± 8	60 ± 10	<0.0001
Sex (M/F)	12/11	11/12	0.77
Weight (kg)	91 ± 11	80 ± 9	<0.0001
Height (cm)	167 ± 14	169 ± 5	0.64
BMI (kg/m^2^)	32 ± 4	28 ± 3	<0.0001
Systolic BP (mmHg)	132 ± 8	122 ± 8	0.001
Diastolic BP (mmHg)	81 ± 7	75 ± 6	0.003
Duration of diabetes (years)	4.5 ± 2.2	N/A	
HbA1c (mmol/mol)	51.2 ± 11.4	35.2 ± 2.2	<0.0001
HbA1c (%)	6.8 ± 1.0	5.4 ± 0.2	<0.0001
Total cholesterol (mmol/L)	4.2 ± 1.0	4.8 ± 0.8	0.01
Triglyceride (mmol/L)	1.7 ± 0.7	1.34 ± 0.6	0.06
HDL cholesterol (mmol/L)	1.1 ± 0.3	1.5 ± 0.4	0.001
LDL cholesterol (mmol/L)	2.2 ± 0.8	2.7 ± 0.87	0.05
CRP (mg/L)	3.0 ± 2.7	5.1 ± 10.3	0.33

BMI, body mass index; BP, blood pressure; HbA1c, HaemoglobinA1c; HDL, high-density lipoprotein; LDL, low-density lipoprotein; CRP, C-reactive protein.

**Table 2:** Baseline values for bone marker proteins (BMPs) for control and patients with type 2 diabetes (T2D). Data presented as mean ± 1 standard deviation of Relative Fluorescent Units (RFUs). *p* values are obtained from repeated measures linear mixed models. * indicates significant difference between Control and T2D at baseline.

	Control		T2D		*p* Value
	Mean	SD	Mean	SD	
Sclerostin	9199	2963	9573	4506	0.63
Dickkopf-related protein 1	18,151	8053	28,249	17,076	0.017 *
Glycogen synthase kinase-3 alpha/beta	4024	1092	4143	1192	0.76
Periostin	2894	556	2596	655	0.08
Tumor necrosis factor ligand superfamily member 11	580	291	571	235	0.91
Fibroblast growth factor 23	544	299	420	50	0.026 *
Sphingosine kinase 1	1802	717	1967	808	0.51
Sphingosine kinase 2	407	109	406	159	0.98
Cathepsin A	5571	1803	8772	4517	0.004 *
Cathepsin B	1362	406	1364	33	0.99
Cathepsin D	857	447	1247	637	0.56
Cathepsin E	290	43	297	53	0.76
Cathepsin G	534	87	643	371	0.16
Cathepsin H	796	282	990	402	0.08
Cathepsin L	1569	711	1415	512	0.38
Cathepsin S	739	169	888	283	0.027 *
Cathepsin Z	3083	668	3607	930	0.032 *
Parathyroid hormone	2684	945	2064	682	0.06
Osteocalcin	1552	925	1919	4234	0.76
Interleukin-1 beta	971	257	959	236	0.92

## Data Availability

Data will be made available upon reasonable request to the corresponding author.

## References

[1] Pietschmann P., Patsch J., Schernthaner G. (2010). Diabetes and Bone. Horm. Metab. Res..

[2] Schwartz A.V. (2009). Impact of Diabetes and Its Treatment on Bone. Clin. Rev. Bone Miner. Metab..

[3] Compston J. (2018). Type 2 Diabetes Mellitus and Bone. J. Intern. Med..

[4] Yang F., Wei F.L., Lang Y., Liu Y.C. (2015). Diabetes Mellitus and Risk of Hip Fractures: A Meta-Analysis. Osteoporos. Int..

[5] Bai J., Gao Q., Wang C., Dai J. (2020). Diabetes mellitus and risk of low-energy fracture: A meta-analysis. Aging Clin. Exp. Res..

[6] Sheu A., Greenfield J.R., White C.P., Center J.R. (2023). Contributors to impaired bone health in type 2 diabetes. Trends Endocrinol. Metab..

[7] Sharma P., Sharma R.K., Gaur K. (2024). Understanding the impact of diabetes on bone health: A clinical review. Metab. Open.

[8] Catalfamo D.L., Britten T.M., Storch D.L., Calderon N.L., Sorenson H.L., Wallet S.M. (2013). Hyperglycemia induced and intrinsic alterations in type 2 diabetes-derived osteoclast function. Oral Dis..

[9] Pierce J., Begun D., Westendorf J., McGee-Lawrence M. (2019). Defining osteoblast and adipocyte lineages in the bone marrow. Bone.

[10] Almutlaq N., Neyman A., DiMeglio L.A. (2021). Are Diabetes Microvascular Complications Risk Factors for Fragility Fracture?. Curr. Opin. Endocrinol. Diabetes Obes..

[11] Douglas I., Evans S., Pocock S.J., Smeeth L. (2009). The Risk of Fractures Associated With Thiazolidinediones: A Self-Controlled Case-Series Study. PLoS Med..

[12] Betteridge D.J. (2011). Thiazolidinediones and Fracture Risk in Patients With Type 2 Diabetes. Diabet. Med..

[13] Freire L.B., Brasil-Neto J.P., da Silva M.L., Miranda M.G.C., de Mattos Cruz L., Martins W.R., da Silva Paz L.P. (2024). Risk factors for falls in older adults with diabetes mellitus: Systematic review and meta-analysis. BMC Geriatr..

[14] Hidayat K., Du X., Wu M., Shi B.M. (2019). The Use of Metformin, Insulin, Sulphonylureas, and Thiazolidinediones and the Risk of Fracture: Systematic Review and Meta-analysis of Observational Studies. Obes. Rev..

[15] Napoli N., Chandran M., Pierroz D.D., Abrahamsen B., Schwartz A.V., Ferrari S.L. (2017). Mechanisms of diabetes mellitus-induced bone fragility. Nat. Rev. Endocrinol..

[16] Clowes J.A., Robinson R.T., Heller S.R., Eastell R., Blumsohn A. (2002). Acute changes of bone turnover and PTH induced by insulin and glucose: Euglycemic and hypoglycemic hyperinsulinemic clamp studies. J. Clin. Endocrinol. Metab..

[17] Kachroo S., Kawabata H., Colilla S., Shi L., Zhao Y., Mukherjee J., Iloeje U., Fonseca V. (2015). Association between hypoglycemia and fall-related events in type 2 diabetes mellitus: Analysis of a U.S. commercial database. J. Manag. Care Spec. Pharm..

[18] Hidayat K., Fang Q.-L., Shi B.-M., Qin L.-Q. (2021). Influence of glycemic control and hypoglycemia on the risk of fracture in patients with diabetes mellitus: A systematic review and meta-analysis of observational studies. Osteoporos. Int..

[19] Xing B., Yu J., Zhang H., Li Y. (2023). RANKL inhibition: A new target of treating diabetes mellitus?. Ther. Adv. Endocrinol. Metab..

[20] Ferrari S., Akesson K., Al-Daghri N., Biver E., Chandran M., Chevalley T., Josse R., Kendler D., Lane N., Makras P. (2025). Bone microstructure and TBS in diabetes: What have we learned? A narrative review. Osteoporos. Int..

[21] Masuhara M., Sato T., Hada N., Hakeda Y. (2009). Protective protein/cathepsin A down-regulates osteoclastogenesis by associating with and degrading NF-kappaB p50/p65. J. Bone Miner. Metab..

[22] Frørup C., Jensen M.H., Haupt-Jorgensen M., Buschard K., Størling J., Pociot F., Fløyel T. (2024). Elevated Cathepsin S Serum Levels in New-Onset Type 1 Diabetes and Autoantibody-Positive Siblings. Diabetes.

[23] Yasuda Y., Kaleta J., Brömme D. (2005). The role of cathepsins in osteoporosis and arthritis: Rationale for the design of new therapeutics. Adv. Drug Deliv. Rev..

[24] Dera A.A., Ranganath L., Barraclough R., Vinjamuri S., Hamill S., Barraclough D.L. (2019). Cathepsin Z as a novel potential biomarker for osteoporosis. Sci. Rep..

[25] Elhadad M.A., Jonasson C., Huth C., Wilson R., Gieger C., Matias P., Grallert H., Graumann J., Gailus-Durner V., Rathmann W. (2020). Deciphering the Plasma Proteome of Type 2 Diabetes. Diabetes.

[26] Duan P., Bonewald L.F. (2016). The role of the wnt/β-catenin signaling pathway in formation and maintenance of bone and teeth. Int. J. Biochem. Cell Biol..

[27] Pinzone J.J., Hall B.M., Thudi N.K., Vonau M., Qiang Y.W., Rosol T.J., Shaughnessy J.D. (2009). The role of Dickkopf-1 in bone development, homeostasis, and disease. Blood.

[28] Lattanzio S., Santilli F., Liani R., Vazzana N., Ueland T., Di Fulvio P., Formoso G., Consoli A., Aukrust P., Davì G. (2014). Circulating dickkopf-1 in diabetes mellitus: Association with platelet activation and effects of improved metabolic control and low-dose aspirin. J. Am. Heart Assoc..

[29] Guo Y.C., Yuan Q. (2015). Fibroblast growth factor 23 and bone mineralisation. Int. J. Oral Sci..

[30] Rhee Y., Bivi N., Farrow E., Lezcano V., Plotkin L.I., White K.E., Bellido T. (2011). Parathyroid hormone receptor signaling in osteocytes increases the expression of fibroblast growth factor-23 in vitro and in vivo. Bone.

[31] Atkin A.S., Moin A.S.M., Nandakumar M., Al-Qaissi A., Sathyapalan T., Atkin S.L., Butler A.E. (2021). Impact of severe hypoglycemia on the heat shock and related protein response. Sci. Rep..

[32] Starup-Linde J., Lykkeboe S., Handberg A., Vestergaard P., Høyem P., Fleischer J., Hansen T.K., Poulsen P.L., Laugesen E. (2021). Glucose variability and low bone turnover in people with type 2 diabetes. Bone.

[33] Moin A.S.M., Al-Qaissi A., Sathyapalan T., Atkin S.L., Butler A.E. (2021). Hypoglycaemia in type 2 diabetes exacerbates amyloid-related proteins associated with dementia. Diabetes Obes. Metab..

[34] Suhre K., Arnold M., Bhagwat A.M., Cotton R.J., Engelke R., Raffler J., Sarwath H., Thareja G., Wahl A., DeLisle R.K. (2017). Connecting genetic risk to disease end points through the human blood plasma proteome. Nat. Commun..

[35] Birkett M.A., Day S.J. (1994). Internal pilot studies for estimating sample size. Stat. Med..

